# Effects of Sex and Mild Intrainsult Hypothermia on Neuropathology and Neural Reorganization following Neonatal Hypoxic Ischemic Brain Injury in Rats

**DOI:** 10.1155/2016/2585230

**Published:** 2016-03-03

**Authors:** Amanda L. Smith, Ted S. Rosenkrantz, R. Holly Fitch

**Affiliations:** ^1^Department of Psychology, Behavioral Neuroscience Division, The University of Connecticut, 406 Babbidge Road, Storrs, CT 06269, USA; ^2^Department of Pediatrics/Neonatology, The University of Connecticut Health Center, 263 Farmington Avenue, Farmington, CT 06030, USA

## Abstract

Hypoxia ischemia (HI) is a recognized risk factor among late-preterm infants, with HI events leading to varied neuropathology and cognitive/behavioral deficits. Studies suggest a sex difference in the incidence of HI and in the severity of subsequent behavioral deficits (with better outcomes in females). Mechanisms of a female advantage remain unknown but could involve sex-specific patterns of compensation to injury. Neuroprotective hypothermia is also used to ameliorate HI damage and attenuate behavioral deficits. Though currently prescribed only for HI in term infants, cooling has potential intrainsult applications to high-risk late-preterm infants as well. To address this important clinical issue, we conducted a study using male and female rats with a postnatal (P) day 7 HI injury induced under normothermic and hypothermic conditions. The current study reports patterns of neuropathology evident in* postmortem* tissue. Results showed a potent benefit of intrainsult hypothermia that was comparable for both sexes. Findings also show surprisingly different patterns of compensation in the contralateral hemisphere, with* increases* in hippocampal thickness in HI females contrasting* reduced* thickness in HI males. Findings provide a framework for future research to compare and contrast mechanisms of neuroprotection and postinjury plasticity in both sexes following a late-preterm HI insult.

## 1. Introduction

A common brain insult, associated with preterm birth (<37 weeks of gestational age (GA); [1, 2]), very low birth weight (VLBW; <1500 grams), and term birth complications (e.g., cord prolapse and cord asphyxia [3, 4]), involves a drop in blood and/or oxygen flow to the brain (hypoxia ischemia; HI). In preterm infants, the vulnerability of the developing brain plays a pivotal role in the etiology of HI, with fragility of the neurovascular system leading to increased risk of hemorrhagic and nonhemorrhagic (ischemic) brain injuries [1, 3, 5–7]. In turn, contemporary premature cohorts often exhibit mild/diffuse forms of injury that include grey matter damage in areas such as the cortex and hippocampus and white-matter tissue loss characteristic of diffuse periventricular leukomalacia (PVL; e.g., tissue loss in corpus callosum and internal capsule [8, 9]). While the brain is highly vulnerable in the preterm infant, the plasticity of the developing brain simultaneously provides a prime target for neural reorganization, prompting further study of brain injuries specific to this unique population of infants. Compensatory mechanisms could offset severe tissue loss (as would be seen in adults with comparable HI events) and instead lead to more subtle anomalies.

Though obvious sex differences characterize development (i.e., delays in male fetal development that may lead to prolonged vulnerability to brain injury in the neonatal period) and are also reported in behavioral outcomes following an HI insult (in both clinical studies and animal models [10–15]), clinicians currently implement identical neuroprotective regimens (hypothermia or “cooling”) for both male and female term infants with hypoxic ischemic encephalopathy (HIE). “Cooling” involves head temperature reductions of 1 to 6°C during/following HI (via head or full body cooling) and has been shown to decrease the metabolic rate of cells, reduce blood flow in the brain, decrease the rate of ATP consumption, and eventually reduce the downstream consequences of dependence on the highly inefficient process of anaerobic metabolism that causes apoptotic cell loss [16–19]. Though demonstrably effective in term infants with mild-to-moderate HIE [20–26], large-scale cooling trials typically* do not* report outcomes as a function of sex. Moreover, although hypothermia has been primarily used in term infants, evidence suggests that at-risk late-preterm infants may also benefit from reduced body temperatures (though parameters may differ). We argue that these important variables need to be investigated further, given the paucity of animal studies examining the effects of cooling in a preterm rodent HI model, coupled with a small but important set of case studies implementing cooling in late-preterm infants [27, 28]. Accumulated questions regarding differential cooling effects as a function of sex and age motivated an exploration of prophylactic (intrainsult) hypothermia in a model of preterm brain injury in male and female rats.

Previously, our lab and others have used the Rice-Vannucci model of induced P7 HI injury in rats to study neuropathologic and behavioral outcomes and to explore the neuroprotective effect of hypothermia and other experimental agents (see [29] for review of neuroprotective agent). Though the P7 HI rat was classically thought to model term infants with HIE, it is now thought to more accurately model insults seen in the late-preterm infant (GW 34–36; this new interpretation is reflected in updates to the website translatingtime.net, in which the Finlay group has calculated age comparisons between species (see [30])). Corresponding results confirm both neuropathologies and behavioral deficits typical of late-preterm HI insult in a P7 HI rat model [31, 32]. Interestingly, in a recent study we have shown that mild temperature reduction during an HI insult led to “task-specific” benefits on a battery of behavioral tasks that generally favored females [33]. This result suggests that intrainsult hypothermia may offer regionally specific protection in a late-preterm model, with differential patterns of behavioral benefit that overall favor females [33]. The paucity of data led us to undertake an additional assessment of neuropathological indices following intrainsult hypothermia in a P7 HI model and to ascertain whether benefits (if seen) would be comparable in males and females.

The current study was designed to address these aims. Specifically, we* first* sought to assess whether lower body temperatures during hypoxia would ameliorate brain injury in general, using a late-preterm (P7 HI) model.* Second*, we sought to evaluate any neuropathologic beneficial effect of intrainsult hypothermia in male and female rats, given evidence of sex differences on a battery of behavioral tasks in these same animals (see [33] for behavioral details). To accomplish our first two aims, gross volumes of grey and white matter areas (as typically affected by an HI insult) were assessed in adulthood. These included cortex, hippocampus, internal capsule, corpus callosum, and lateral ventricles. In addition (for a* third* aim), given that we have previously shown sex differences in outcomes on a memory task following P7 HI, we sought to more closely analyze the “uninjured” (contralateral) hippocampus. Specifically, we wanted to determine whether some form of compensation might occur following an HI insult. Compensatory mechanisms of the hemisphere contralateral to the ischemic insult have not been fully investigated, including whether this hemisphere sustains subtle hypoxic damage similar to the ipsilateral hemisphere or displays compensatory alterations reflecting the plasticity of the developing brain. To address this third aim, different regions of the hippocampal cell layers in the contralateral hemisphere were assessed for thickness. We specifically sought evidence of neural compensation that could explain better performance in females as compared to males on a post-HI-insult memory task.

Given prior findings, we hypothesized that (1) intrainsult hypothermia would ameliorate (reduce) indices of brain damage in a preterm model of HI; (2) if intrainsult hypothermia was indeed beneficial, sex-specific benefits in favor of female HI rats might be seen; and (3) if the benefits of intrainsult hypothermia were in fact comparable by sex, then the contralateral hippocampus might show evidence of compensation to HI injury (e.g., thicker cell layers) that would be more prominent in female HI injured rats. Results of the current study* confirmed* the efficacy of intrainsult hypothermia in a late-preterm model of HI, as measured by neuropathology. Moreover, neuroprotection was comparable in both sexes, but sex differences in potential compensatory mechanisms were apparent in the contralateral hippocampus.

## 2. Methods

### 2.1. Methodological Background: Postmortem Tissue

Brain tissue from a prior study of male and female HI injured rats (for which we previously reported behavioral outcomes) was used in the current study (see [33] for detailed methods). In brief, subjects were male (*n* = 31) and female (*n* = 31) Wistar rats born to time-mated dams (Charles River Laboratories) shipped to the University of Connecticut Bousfield vivarium on embryonic (E) day 5. At birth (approximately E22), pups were culled to litters of 5 females and 5 males. On P7, pups were randomly selected for HI or sham procedure and assigned to the hypothermic or normothermic group. HI animals were anesthetized with isoflurane (2.5%), a vertical incision was made on the neck, and the right common carotid artery was cauterized to restrict blood flow to the right hemisphere (all possible steps were taken to avoid animal suffering during all surgical procedures, with oversight and approval from the University of Connecticut Institutional Animal Care and Use Committee (IACUC)). Sham animals received a similar procedure without artery cauterization. Following surgery, normothermic animals (HI and sham) were placed in a temperature-controlled incubator with a warming lamp, while hypothermic animals (HI and sham) were placed in an unheated holding container with a warming lamp. During the 2-hour hypoxia period, HI normothermic animals were placed in an airtight temperature-controlled container subjected to 8% oxygen (balanced with nitrogen). HI hypothermic animals were placed in a similar airtight container positioned under a warming lamp and were also subjected to 8% oxygen (balanced with nitrogen). Sham animals were placed in similar containers with similar temperature environments. Temperatures were taken of all animals to ensure each group was kept at normothermic or hypothermic conditions. For analyses, sham hypothermic and normothermic animals were pooled for each sex.

### 2.2. Prior Behavioral Testing

Behavioral testing began on P30 and continued until P83. All animals were tested on a rotarod task to assess motor coordination and learning, an auditory discrimination task to assess auditory processing, and a Morris water maze (MWM) and nonspatial water maze task to assess learning and memory. Detailed methodology for each behavioral task is available elsewhere (see [33]), but the overall findings from each behavioral task are outlined in Table 1.

### 2.3. Raw Volume Measurement (See Table 2)

Following the completion of behavioral testing, animals were anesthetized with ketamine (100 mg/kg) and xylazine (15 mg/kg) and transcardially perfused with 0.9% saline solution followed by 10% buffered formalin. Brains were removed from the skull and placed in 10% formalin until slicing began. A Leica VT1000 S vibratome was used (60 *μ*m), with every other section mounted on a chrome-alum subbed slide. All brains were stained using the Nissl staining procedure. For gross volumetric measures, Stereo Investigator Microbright field software and an Axio 2 Zeiss Microscope were used. Volumes were quantified using 2.5x magnification with Cavalieri's Estimator software and a grid overlay. The fewest number of sections was counted to achieve a coefficient of error less than 0.05 (stereological validity) for each area analyzed (usually between 8 and 10 sections per brain area), and every third mounted section was assessed. For the cortex, sections were analyzed starting at approximately Bregma 1.70 mm to approximately Bregma −4.16 mm. For the hippocampus, sections were analyzed starting at approximately Bregma −2.30 mm to approximately Bregma −6.04. For the internal capsule, sections were analyzed beginning at Bregma −1.40 mm to approximately Bregma −4.52 mm. For the corpus callosum, sections were analyzed beginning at approximately Bregma 1.20 mm to approximately Bregma −0.92 mm. Finally, for the lateral ventricles, sections were analyzed beginning at approximately Bregma 1.60 mm to approximately Bregma −1.30 mm. All measures are approximations due to the variability in the quality of brain tissue and were performed blind to Treatments.

### 2.4. Raw Pyramidal Cell Thickness Measures (See Table 3)

To assess cell layer thickness, the hippocampus was divided into 4 sections (CA1, medial CA3 (within the dentate gyrus), lateral CA3 (outside of dentate gyrus), and dentate gyrus). The borders of the hippocampal layers of each hemisphere were traced on an Axio 2 Zeiss microscope under 20x, from one representative section of each subject, at Bregma −3.14 mm. Boundaries of the layers were determined by only including pyramidal cells (or granule cells in the dentate gyrus) in contact with one another in each band, essentially creating a “line of best fit” for each border. Once the entire hippocampus was traced, measurements from each section (CA1, medial CA3, lateral CA3, and dentate gyrus) were taken by drawing a line from the bottom border to the top border of the cell layer. This provides a length (in *μ*m) from the top to the bottom of the cell layer. Five representative lengths were taken from CA1 and the dentate gyrus, and three representative lengths were taken from both regions of CA3. Measurements were taken from equidistant points within each hippocampal section assessed. Averages from each subregion were calculated and from these values, an overall average thickness measure was calculated for each subject in each hemisphere.

### 2.5. Statistics

For volumetric measures, individual repeated measures ANOVAs were performed to compare ipsilateral and contralateral raw volumes of cortex, hippocampus, ventricles, internal capsule, and corpus callosum. Variables included Sex (2 levels), Treatment (3 levels; HI normothermic, HI hypothermic, and sham; between subject variables), and Hemisphere (2 levels; repeated variable). Further repeated measures ANOVAs were performed for each sex separately using 3 Treatment groups (HI hypothermic, HI normothermic, and sham), as well as individual one-way ANOVAs performed for each hemisphere in each sex. To assess effects within each HI group separately, paired samples *t*-tests were performed comparing ipsilateral and contralateral hemisphere volume for each structure. This analysis was performed including both sexes, as well as each sex separately. Results are presented in Table 2.

To specifically assess overall tissue loss in all 5 brain areas, a percentage was calculated from ipsilateral and contralateral volumes (contralateral volume – ipsilateral volume/total volume *∗* 100) for each area. For the ventricles, this was calculated as a percent increase. These values were used to assess Treatment effects by performing univariate ANOVAs for each brain area, using Injury (2 levels; HI and sham), Temperature (2 levels; hypothermic and normothermic), and Sex (2 levels; male and female) as variables. Independent samples *t*-tests comparing all HI normothermic and all HI hypothermic animals were also performed, to investigate hypothermia's beneficial effect on brain damage following HI (performed for males and females separately).

For pyramidal cell layer thickness, averages were calculated from delineating boundaries histologically identified for each section (CA1, medial CA3, lateral CA3, and dentate gyrus), for each subject. Individual one-way ANOVAs were performed to assess Treatment effects in each hippocampal section of both hemispheres, for males and females separately. Due to the small *n* in the HI normothermic groups for the ipsilateral hemisphere analyses (resulting from gross tissue damage), an overall repeated measures ANOVA was performed using Area (4 levels) and Treatment (6 levels) to assess the pattern of damage and/or compensation in the* contralateral hemisphere only*. In order to investigate the effect of HI in the contralateral hemisphere independent of temperature modulation, another repeated measures ANOVA was performed using Area (4 levels) and Treatment (4 levels; male and female HI normothermic and male and female sham). This analysis was again performed for each sex (HI normothermic and sham) separately. Finally, an overall contralateral pyramidal cell thickness measure was calculated from means of each hippocampal region. These values were used to perform an ANOVA with Sex (2 levels) and Treatment (HI normothermic and sham) as variables. Further independent samples *t*-tests were performed in each sex separately to assess sex-specific Treatment effects.

## 3. Results

### 3.1. Volumetric Analyses

In brief, results from analyses of raw volumetric measures demonstrated (1) a significant reduction in* all* ipsilateral hemisphere volumetric brain measures in both sexes following HI (with a significant increase in the ipsilateral lateral ventricle) and (2) a significant benefit from intrainsult hypothermia in preserving volumes in all regions, in both sexes, following HI (though not to the level of shams). These data are captured in pathology indices analyzed below, but effects for specific regional volumetric comparisons are provided, along with absolute mean values, in Table 2 (volumes and standard errors for each brain area analyzed).

### 3.2. Percent Damage Analyses (Treatment Effects; Figures 1 and 2)

**Figure 1:**
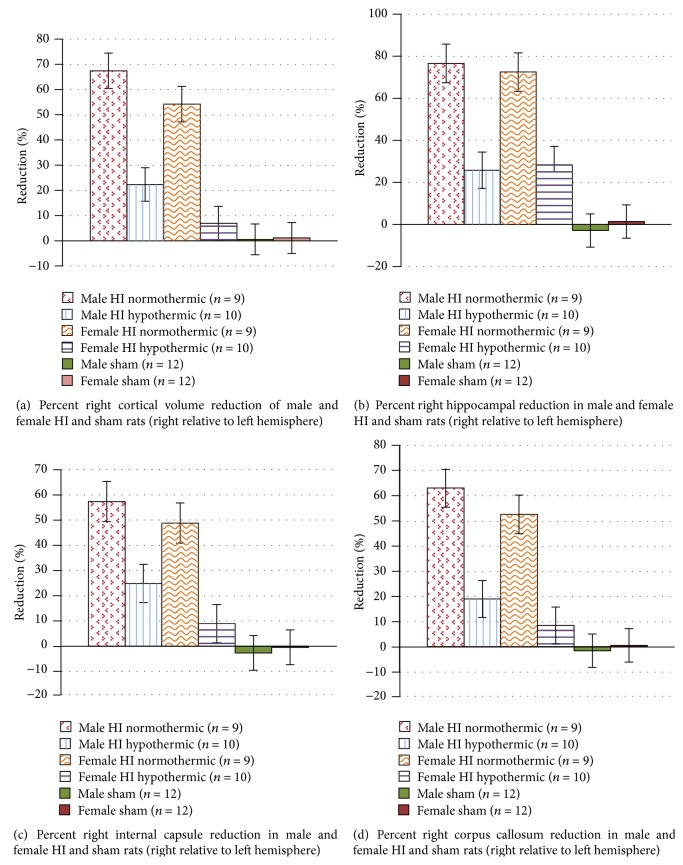
(a) A 2 (Sex) × 2 (Injury) × 2 (Temperature) ANOVA revealed a significant Injury (*p* < 0.05) and Temperature (*p* < 0.05) effect, as well as an Injury × Temperature interaction (*p* < 0.05) and a trend for a Sex × Injury interaction (*p* = 0.19). An independent samples *t*-test between HI normothermic and HI hypothermic animals revealed a significant effect (*p* < 0.05) and a similar effect was seen when *t*-tests were performed for males (*p* < 0.05) and females (*p* < 0.05) separately. (b) A 2 (Sex) × 2 (Injury) × 2 (Temperature) ANOVA revealed a significant Injury (*p* < 0.05) and Temperature (*p* < 0.05) effect, as well as an Injury × Temperature interaction (*p* < 0.05). An independent samples *t*-test between HI normothermic and HI hypothermic animals revealed a significant effect (*p* < 0.05) and a similar effect was seen when *t*-tests were performed for males (*p* < 0.05) and females (*p* < 0.05) separately. (c) A 2 (Sex) × 2 (Injury) × 2 (Temperature) ANOVA revealed a significant Injury (*p* < 0.05) and Temperature (*p* < 0.05) effect, as well as an Injury × Temperature interaction (*p* < 0.05). An independent samples *t*-test between HI normothermic and HI hypothermic animals revealed a significant effect (*p* < 0.05) and this pattern was also seen in females (*p* < 0.05) but not in males (*p* > 0.05). (d) A 2 (Sex) × 2 (Injury) × 2 (Temperature) ANOVA revealed a significant Injury (*p* < 0.05) and Temperature (*p* < 0.05) effect, as well as an Injury × Temperature interaction (*p* < 0.05). An independent samples *t*-test between HI normothermic and HI hypothermic animals revealed a significant effect (*p* < 0.05) and this pattern was also seen in females (*p* < 0.05) but not in males (*p* > 0.05).

**Figure 2:**
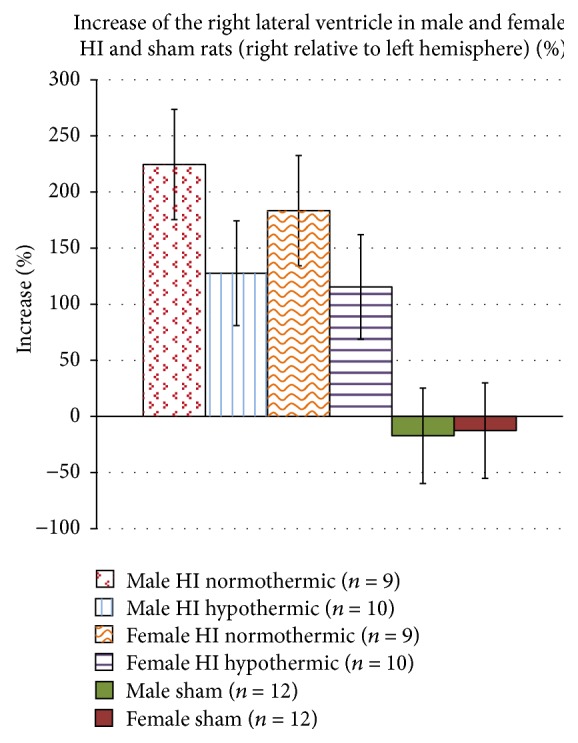
A 2 (Sex) × 2 (Injury) × 2 (Temperature) ANOVA revealed a significant Injury effect (*p* < 0.05). Independent samples *t*-tests did not yield significant effects.

Following damage calculations described above, an ANOVA using Sex (2 levels; male and female), Injury (2 levels; HI and sham), and Temperature (2 levels; normothermic and hypothermic) was performed to assess* cortical* percent damage. Effects revealed significant Injury [*F*(1,54) = 43.374, *p* < 0.05] and Temperature [*F*(1,54) = 15.462, *p* < 0.05] main effects, as well as a significant Injury × Temperature interaction [*F*(1,54) = 18.551, *p* < 0.05]. These reflect increased damage in HI; decreased damage in hypothermic animals; and a specific decrease in damage in HI hypothermic animals (resp.). A trend for a significant Sex × Injury interaction was also seen [*F*(1,54) = 1.756, *p* = 0.19], suggesting more HI damage in males. When assessing* hippocampal, internal capsule,* and* corpus callosum* percent reduction, significant Injury and Temperature effects (resp.) were seen for each area (hippocampus: [*F*(1,54) = 49.848, *p* < 0.05], [*F*(1,54) = 9.91, *p* < 0.05]; internal capsule: [*F*(1,54) = 33.32, *p* < 0.05], [*F*(1,54) = 7.228, *p* < 0.05]; corpus callosum: [*F*(1,54) = 35.321, *p* < 0.05], [*F*(1,54) = 12.674, *p* < 0.05]), reflecting the same patterns as seen in cortex. We also saw significant Surgery Injury × Temperature interactions (hippocampus: [*F*(1,54) = 11.235, *p* < 0.05]; internal capsule: [*F*(1,54) = 9.117, *p* < 0.05]; corpus callosum: [*F*(1,54) = 13.398, *p* < 0.05]), again indicating that in all areas, the percent reduction was significantly less in hypothermic as compared to normothermic HI animals (see Figure 1). Finally, an ANOVA using ventricular percent increase values revealed a significant Injury effect ([*F*(1,54) = 20.59, *p* < 0.05], see Figure 2), with larger volumes in HI subjects.

Independent samples *t*-tests to compare percent reduction for HI normothermic versus HI hypothermic animals (across sex) revealed significant effects when looking at the cortex [*t*(36) = 5.229, *p* < 0.05], hippocampus [*t*(36) = 4.399, *p* < 0.05], internal capsule [*t*(36) = 3.709, *p* < 0.05], and corpus callosum [*t*(36) = 4.666, *p* < 0.05]. Results showed that HI normothermic animals had a larger percent reduction in all regions as compared to HI hypothermic animals. No significant effects were seen when performing independent samples *t*-tests for ventricular percent increase. Additional independent samples *t*-tests were performed for each sex separately for each brain area and revealed similar effects for males and females when assessing cortical percent reduction ([*t*(17) = 3.184, *p* < 0.05] and [*t*(17) = 4.603, *p* < 0.05], resp.), hippocampal percent reduction ([*t*(17) = 2.928, *p* < 0.05] and [*t*(17) = 3.195, *p* < 0.05], resp.), and corpus callosum percent reduction (*t*(17) = 2.979, *p* < 0.05] and [*t*(17) = 3.648, *p* < 0.05], resp.). These findings confirm that HI normothermic animals had more damage than HI hypothermic animals, across sex, indicating that intrainsult hypothermia was in fact beneficial for both sexes in this model of preterm HI injury. For the internal capsule, an independent samples *t*-test revealed a significant difference between HI normothermic and HI hypothermic females [*t*(17) = 3.648, *p* < 0.05] but not for males. No significant effects were seen when assessing percent increase for the ventricles (Figure 2).

### 3.3. Pyramidal Cell Layer Thickness Measures

**Figure 3:**
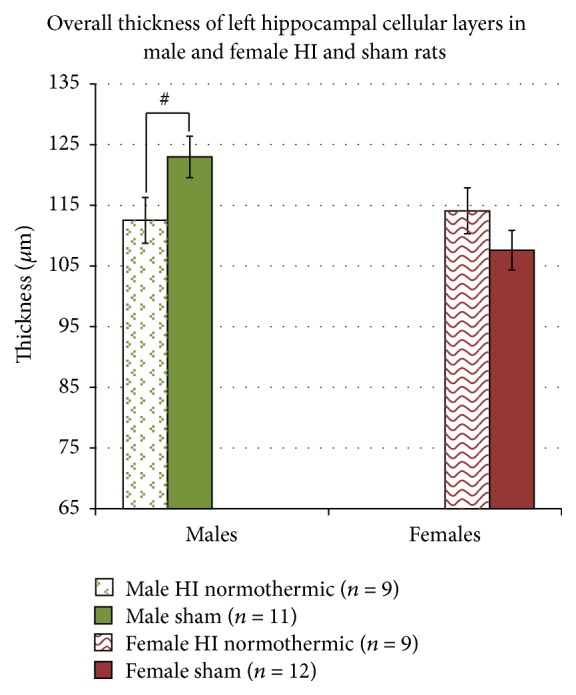
A 2 (Sex) × 2 (Treatment) ANOVA revealed a trend for a significant Sex effect (*p* = 0.06) and a significant Sex × Treatment interaction (*p* < 0.05). Individual independent samples *t*-tests for each sex separately revealed a trend for HI male normothermic animals to have significantly* thinner* hippocampal cellular layer thickness (^#^ *p* = 0.077), but HI female normothermic animals were revealed to have thicker hippocampal cell layers.

Individual one-way ANOVAs were initially performed to assess Treatment effects (3 levels; HI normothermic, HI hypothermic, and sham) for each sex in the ipsilateral hippocampal area (CA1, medial CA3, lateral CA3, and dentate gyrus). For both sexes, no significant Treatment effects were found in any hippocampal area, probably due to the very small *n* for the HI normothermic group in the ipsilateral hemisphere for each sex. We then performed a 4 (Area) × 2 (Sex) × 3 (Treatment; sham, HI normothermic, and HI hypothermic) repeated measures ANOVA using just the measurements from the contralateral hemisphere and found a significant effect of Area [*F*(3,156) = 343.947, *p* < 0.05], a near-significant Sex effect [*F*(1,52) = 3.807, *p* = 0.056], and trend for a significant Treatment × Sex interaction [*F*(2,52) = 2.473, *p* = 0.09] (see Table 3 for averages and standard errors for each subregion for all groups). To assess HI effects independent of temperature modulation, a 4 (Area) × 2 (Sex) × 2 (Treatment; HI normothermic and sham) repeated measures ANOVA revealed a significant Sex × Treatment interaction [*F*(1,37) = 5.621, *p* < 0.05], as well as a marginally significant Sex effect [*F*(1,37) = 3.748, *p* = 0.06] and Area × Sex interaction [*F*(1,37) = 2.996, *p* = 0.09]. Two ANOVAs using Treatment (2 levels, HI normothermic, and sham) × Area (4 levels) to assess the contralateral hemisphere of each sex separately, revealed a near-significant Treatment effect in males [*F*(1,18) = 3.511, *p* = 0.07] and a trend in females [*F*(1,19) = 2.064, *p* = 0.16]. An overall thickness measure of the contralateral hemisphere was then calculated using the averages of the 4 hippocampal layers. An ANOVA using these contralateral hemisphere averages with Sex (2 levels) and Treatment (2 levels) as variables revealed a near-significant Sex effect [*F*(1,37) = 3.748, *p* = 0.06] and a significant Sex × Treatment interaction [*F*(1,37) = 5.621, *p* < 0.05], indicating* a significantly different neurodevelopmental response to HI injury in the contralateral-hippocampal hemisphere for males and females*. Independent samples *t*-tests for each sex revealed a near-significant Treatment effect in males [*t*(18) = −1.874, *p* = 0.07], with HI animals having* thinner *contralateral-hippocampal values, and a trend in females [*t*(19) = 1.437, *p* = 0.16], with HI animals having* thicker* contralateral-hippocampal values (see Figure 3).

## 4. Discussion

The current study utilized a well-established rodent model of late-preterm HI injury to investigate benefits of intrainsult hypothermia as measured by neuropathology. Moreover, sex differences in protective benefit of intrainsult hypothermia were also investigated. Finally, based on behavioral findings described in a prior publication [33], we further sought to investigate subtle patterns of brain damage—particularly in cell layers of hippocampal subregions—in response to HI. Previous studies from our lab provided a framework for the current study, by demonstrating sex differences on an array of behavioral paradigms (most notably in the memory domain) following an experimentally induced HI insult in P7 rats (modeling late-preterm brain injury [15, 33]). Surprisingly, these sex differences in behavioral outcomes were* not* coupled to detectable sex-specific patterns of brain injury [15]. In fact, male and female HI injured rats displayed strikingly similar degrees of brain damage in the hippocampus, cortex, and ventricles. Moreover, when investigating the protective effect of intrainsult hypothermia on behavioral deficits, female HI rats also displayed a slightly more robust protective effect compared to males [33]. The current data replicate previously reported HI damage in the hippocampus, cortex, and ventricles [15, 34–36], as well as significant brain damage in the corpus callosum and internal capsule associated with P7 HI. And again, these indices were comparable for male and female HI rats. In addition, we obtained* new* evidence that damage was uniformly attenuated in HI animals treated with intrainsult hypothermia—reaffirming (together with previously reported behavioral benefits) that a mild prophylactic hypothermic intervention might be translatable to high-risk late-preterm infants (both males and females). Our final key novel finding was that the hippocampus contralateral to the HI injury showed a Sex × Injury interaction in cell layer thickness, which can be taken as evidence of a sex difference in neural response to contralateral injury. The contrast between increased thickness in females and decreased thickness in males could account for a female advantage on memory-based behavioral tasks, as previously reported following intrainsult hypothermia in a P7 HI model [33]. This finding could have key importance in understanding clinical reports of a female advantage in outcomes in general following preterm birth [15, 37, 38] and also prompts further examination of application and mechanisms of neuroprotection in the late-preterm population, specifically as a function of sex.

### 4.1. Neuropathology of HI in the Late Preterm Neonate/P7 HI Rat Model

Prior clinical and animal studies investigating the neuropathology of HI insult show a relatively ubiquitous phenotype of brain damage in premature infants. White matter damage predominates, due to the vulnerability created by the timing of oligodendrocyte precursors actively proliferating and differentiating [39], and it is not surprising that the majority of preterm infants with HI show later white matter anomalies. Animal models of P7 HI (a late-preterm-equivalent injury) and other animal models of injury also reveal significant white matter loss, including reduced corpus callosum area [36, 40, 41]. Thus although larger animal models (i.e., sheep) are optimal for modeling white matter HI insult (due to the abundance of myelinated fiber tracts [42]), rodent models of preterm brain injury also replicate these features [40, 43, 44]. White matter injury was replicated in the current study, with both male and female HI animals showing comparable decreases in the volume of corpus callosum and internal capsule (see Table 2 and Figure 1). Finally, in addition to white matter damage, HI animals displayed a significant loss of grey matter in areas including cortex and hippocampus—thus replicating findings from both clinical human data and animal HI models [3, 15, 34, 35, 43, 44].

### 4.2. Neuropathology and Hypothermia Rescue following HI

As noted above, all HI subjects showed white matter loss, but these losses were significantly less severe in hypothermic as compared to normothermic HI animals (both males and females; see Figure 1). Thus our data provide anatomic verification of the protective effect of intrainsult hypothermia in a model of late-preterm HI injury. These findings are consistent with previous studies on the protective effect of hypothermia on oligodendrocyte precursor cell death, as well as subsequent maturation and myelin repair (*in vivo* and* in vitro* [45]). Moreover, although both HI normothermic and HI hypothermic males and females sustained significant damage to grey matter (cortex, hippocampus), hypothermic animals had significantly less grey matter damage than normothermic subjects—again affirming the translational potential of neuroprotection via hypothermia in preterm infants (Figures 1 and 2). These findings are consistent with general models of hypothermia as a mechanism to slow apoptosis and prevent cell death [19, 46, 47].

### 4.3. Sex Differences in HI Neuropathology

Grey and white matter analyses from the current study indicate that despite differences in behavioral outcomes, HI males and females had similar underlying patterns of gross HI damage, as well as similar anatomic response to intrainsult hypothermia rescue. The discontinuity between sex differences in behavioral outcomes and* comparable* pathology across sexes suggests that gross morphology may not be an optimal sole index of outcome for preterm infants. Even hypothermia studies suggest the equation for injury and outcome is not one-to-one. For example, in one study using a P10 HI model, the hippocampus was so severely damaged in both hemispheres that hypothermia was unable to provide protection, despite rescuing tissue loss in other areas (e.g., cortex [32]). In other animal studies, hypothermia implemented shortly after an HI insult was significantly protective to the hippocampus, regardless of severity of injury (primarily seen in the CA1 region [48–51]). Although sex differences in response to intrainsult hypothermia have not been thoroughly investigated, the discontinuity we observed may shed light on the importance of assessing more detailed neuropathological indices (in addition to gross pathology) in order to effectively predict outcomes for both sexes. These factors would certainly include, but not rely exclusively on, gross macroscopic* in vivo* pathology indices.

### 4.4. Postinjury Compensatory Reorganization in Females

To further examine our findings (see Table 1 for summary [33]), we explored the possibility of an innate female protection extending beyond gross pathology. As seen in Figure 1, the hippocampus sustained the highest percentage of HI damage (with male and female HI normothermic and HI hypothermic animals showing almost identical scores), and this severe tissue loss likely reflects enhanced susceptibility to glutamate-mediated cell death in this region [52–54]. Despite the comparable and severe HI induced loss, we previously failed to show significant deficits in HI females on a task reliably associated with hippocampal function—the Morris water maze [15]. As such, we decided to take a closer look at pyramidal cell layers of the CA1 and CA3 subregions of the hippocampus and granule cell layer of the dentate gyrus in order to attempt to quantify reorganization/compensation in the left hippocampus, after severe HI injury in the right. However, these measures did* not* reveal a robust Treatment main effect. Rather, when looking at an overall thickness measure, male and female HI normothermic animals showed* opposite* patterns (as supported by a significant Sex × Injury interaction in mean thickness). Whereas male HI normothermic animals had near-significant reductions in the thickness of the cell layers of the hippocampus, female HI normothermic animals had marginal* increases* in the thickness of the cell layers (see Figure 3). This finding could relate to a previously observed female advantage on a Morris water maze task, indicating that this prior result was not due to sex differences in primary hippocampal damage following HI, but rather due to an enhanced reorganization in the contralateral hemisphere postinsult. In support of this interpretation, another animal study specifically investigating hippocampal damage following HI reported an increase in total dendritic length and dendritic spine density of pyramidal neurons in the left hippocampus (contralateral to the injury), while the ipsilateral hippocampus sustained typical HI damage [55]. Since the sex of animals was not specified, we might infer that both males and females were used, which could mean that compensation in female HI animals may have contributed to the overall findings [55].

In general, the ability of the developing nervous system to reorganize itself following an injury has been explored in human and animal studies, and an elegant body of work on this topic was originally published by Kennard ([56, 57] see [58] for review). More recently, Kolb and colleagues performed cross-age assessments of neural compensation in rats and found that response to loss of cortical tissue varies depending on age at injury, as well as whether lesions were unilateral or bilateral [59, 60]. Another report showed that unilateral cortical lesions in neonatal rats led to structural and functional changes in the remaining hemisphere that were* not* seen after a similar lesion in adults [61]. Findings of similar compensatory changes are reported for preterm infants [62], and in studies of neonatal hemidecortication, where the intact cortex shows increased thickness and reorganized connectivity [63–65].

### 4.5. Hypothermia versus Sex

An interesting conclusion from the current study is that both intrainsult hypothermia and “being female” seem to provide protection from HI insults in a late-preterm model,* but the mechanisms are quite different*. This interpretation is consistent with the fact that no significant differences were seen in the contralateral hippocampus for either* hypothermic* HI males or females. In fact, both sexes showed thickness changes in the direction of, but less than, the same-sex normothermic HI (data not shown, no significant comparisons were found). An important conclusion from this discrepancy could be that because intrainsult hypothermia acts by reducing initial neuropathology, it may* also* reduce postinjury compensatory mechanisms in females. Since hypothermic HI females performed significantly better than HI normothermic females on previously administered behavioral tasks [33], we can infer that the* net* effect for females is still beneficial. However, convergent findings should serve as a “wake-up” call to basic research on HI interventions that ignores the role of sex.

### 4.6. Limitations

As a final note, we recognize the need for several points of caution in translation of the current findings. (1) A unilateral ischemia model, coupled with bilateral hypoxia, does not provide a perfect model for all late-preterm HI events (which tend to be more diffuse and often bilateral). (2) The use of experimental intrainsult hypothermia differs from the current practice of postinsult intervention in HIE term infants (wherein much of our knowledge about neonatal cooling has been obtained). (3) The implementation of cooling in a preterm population characterized by immature thermoregulation and other nonneurologic health concerns remains problematic and tempers incentives for preterm clinical trials. All of these factors must be considered when translating the current findings to a clinical setting. Nonetheless, the findings certainly prompt further study of topics that can improve medical intervention and prognosis for late-preterm infants with HI events, specifically as a function of cooling intervention and patient sex.

## 5. Conclusions

The results of the current study contribute important data to the field of neonatal hypoxic ischemic injury research. Specifically, we found that (1) HI injury induced on P7 leads to long-term brain injury in both grey and white matter in both males and females, and intrainsult hypothermia significantly ameliorates that pathology; (2) intrainsult hypothermia benefits neuropathology equally in both sexes, despite sex differences favoring females in behavioral outcomes; and (3) males and females show significantly different patterns of compensation in the contralateral hippocampus, as measured by thickness of hippocampal cellular layers. Our findings shed light on previously published behavioral data using the same cohort of animals assessed here [33], in which females with P7 HI injury were found to show a greater benefit from intrainsult hypothermia then P7 HI males on several memory tasks, as well as prior sex difference reports in neonatal HI outcomes from our lab. Taken together, results suggest that hypothermia—though currently implemented only for term HIE infants—may also have clinical benefits for some late-preterm infants at high risk for HI events. Overall, the current findings call for further study of the role of sex and hypothermia in response to neonatal HI injury and may encourage clinicians to more carefully consider sex in neonatal neuropathologic research.

## Figures and Tables

**Table 1:** Behavioral findings from Smith et al. [33].

Task	Benefit of hypothermia
Rotarod	Beneficial to females
Silent gap (auditory processing)	Beneficial to males
Morris water maze	Beneficial to females
Nonspatial maze	Beneficial to males and females

**Table 2:** Mean and standard error (SE) for volumetric measures of 6 brain areas in the right and left hemisphere reflecting reductions in the right hemisphere.

	Left hemisphere					Right hemisphere				
	Cortex	Hippocampus	Internal capsule	Corpus callosum	Ventricle	Cortex	Hippocampus	Internal capsule	Corpus callosum	Ventricle
Male HI normothermic (*n* = 9)	144.80 ± 5.52	15.82 ± 1.90	7.22 ± 0.50	8.93 ± 0.68^*∗*^	3.40 ± 0.59	37.35 ± 12.57^*∗*^	3.01 ± 2.15^*∗∗∗*^	2.20 ± 0.61^*∗∗∗*^	3.02 ± 1.10^*∗*^	10.25 ± 1.18^*∗*^
Male HI hypothermic (*n* = 10)	155.02 ± 5.23	16.08 ± 1.80	7.36 ± 0.47	11.22 ± 0.64^*∗∗*^	3.40 ± 0.56	114.32 ± 11.92^*∗∗*^	11.57 ± 2.04^*∗∗∗*^	4.77 ± 0.58^*∗∗∗*^	9.10 ± 1.05^*∗∗*^	5.96 ± 1.12^*∗∗*^
Male sham (*n* = 12)	150.82 ± 4.78	20.16 ± 1.64	7.31 ± 0.43	12.40 ± 0.59	3.96 ± 0.51	149.41 ± 10.88	21.15 ± 1.86^*∗∗∗*^	7.62 ± 0.53^*∗∗∗*^	12.83 ± 0.95	2.87 ± 1.02

Female HI normothermic (*n* = 9)	147.41 ± 5.52	18.94 ± 1.90	7.77 ± 0.50	9.50 ± 0.68^*∗*^	3.46 ± 0.59	54.61 ± 12.57^*∗*^	4.44 ± 2.15^*∗*^	2.96 ± 0.61^*∗*^	4.26 ± 1.01^*∗*^	8.79 ± 1.18^*∗*^
Female HI hypothermic (*n* = 10)	144.61 ± 5.23	15.14 ± 1.80	6.57 ± 0.47	11.76 ± 0.64^*∗∗*^	2.37 ± 0.56	126.76 ± 11.92^*∗∗*^	9.98 ± 2.04^*∗*^	5.54 ± 0.58^*∗∗*^	10.02 ± 1.05^*∗∗*^	4.58 ± 1.11^*∗∗*^
Female sham (*n* = 12)	146.69 ± 4.78	21.03 ± 1.64	7.08 ± 0.43	11.78 ± 0.59	2.30 ± 0.51	143.01 ± 10.88	20.08 ± 1.86	7.16 ± 0.53	11.70 ± 0.95	1.90 ± 1.02

^*∗*^Significant reductions as compared to sham counterparts.

^*∗∗*^Significant differences between HI hypothermic and HI normothermic animals.

^*∗∗∗*^Significant differences between all 3 Treatment groups within each Sex.

**Table 3:** Mean and standard error (SE) for subregions of the left hippocampus.

	Left hippocampal area			
	CA1	CA3 (within dentate gyrus)	CA3 (outside of dentate gyrus)	Dentate gyrus
Male HI normothermic (*n* = 9)	77.278 ± 3.616	165.526 ± 9.037	104.759 ± 6.536	102.571 ± 3.912
Male HI hypothermic (*n* = 9)	75.602 ± 3.616	165.319 ± 9.037	118.022 ± 6.536	96.898 ± 3.912
Male sham (*n* = 11)	89.782 ± 3.271	179.179 ± 8.174	114.924 ± 5.912	108.002 ± 3.538
Female HI normothermic (*n* = 9)	76.800 ± 3.616	172.759 ± 9.037	114.541 ± 6.536	92.238 ± 3.912
Female HI hypothermic (*n* = 8)	76.443 ± 3.835	169.996 ± 9.585	100.108 ± 6.932	88.432 ± 4.149
Female sham (*n* = 12)	77.245 ± 3.132	164.928 ± 7.826	99.681 ± 5.660	88.581 ± 3.388
